# Effects of *Urtica dioica* supplementation on blood lipids, hepatic enzymes and nitric oxide levels in type 2 diabetic patients: A double blind, randomized clinical trial

**Published:** 2016

**Authors:** Alidad Amiri Behzadi, Hamid Kalalian-Moghaddam, Amir Hossein Ahmadi

**Affiliations:** 1 *Young Researcher and Elites Club, North Tehran Branch, Islamic Azad University, Tehran, Iran*; 2 *Department of Physiology, Shahroud University of Medical Sciences, Shahroud, Iran*; 3 *Department of Basic Sciences, Islamic Azad University Damghan Branch, Damghan, Iran*

**Keywords:** *Urticadioica*, *Hydro-alcoholic extract*, *Oxidative stress*, *Type2 diabetes*

## Abstract

**Objective:**

Oxidative stress plays an important role in the development of diabetic complications including metabolic abnormality-induced diabetic micro-vascular and macro-vascular complications. *Urtica dioica L.* (*U. dioica*) has been traditionally used in Iranian medicine as an herbal remedy for hypoglycemic or due to its anti-inflammatory properties. The aim of the present study was to evaluate the effects of hydro-alcoholic extract of *U. dioica* on blood lipids, hepatic enzymes and nitric oxide levels in patients with type 2 diabetes mellitus.

**Materials and Methods:**

50 women with type 2 diabetes participated in this study and were randomly divided into two groups namely, control and intervention groups. Control group received placebo and intervention group received hydro-alcoholic extract of *U. dioica*. Before and after 8 weeks of continuous treatment, some biochemical serum levels including FPG, TG, SGPT, SGOT, HDL, LDL, SOD and NO were measured.

**Results:**

The results indicated that after 8 weeks, in the intervention group, FPG, TG, and SGPT levels significantly decreased and HDL, NO and SOD levels significantly increased as compared to the control group.

**Conclusion:**

Our results encourage the use of hydro-alcoholic extract of *U. dioica* as an antioxidant agent for additional therapy of diabetes as hydro-alcoholic extract of *U. dioica* may decrease risk factors of cardiovascular incidence and other complications in patients with diabetes mellitus.

## Introduction

Diabetes mellitus is one of the most common diseases and hyperglycemia is one of the predisposing factors for oxidative stress (Manohar et al., 2013 ▶; Petal et al., 2012 ▶; Rasheed et al., 2008 ▶). In a healthy individual, there is a balance between antioxidant enzymes and free radical species in the body and an imbalance causes oxidative stress. Free radicals are atoms or molecules with unpaired electron, which are highly active and can damage different tissues in the body (Nazemi et al., 2012 ▶). Therefore, increasing oxidative stress can increase risk factors of cardiovascular incidence and other complications in patients with diabetes mellitus (Rains and Jain, 2011 ▶; Malekirad et al., 2011 ▶). Reactive oxygen species (ROS) such as hydrogen peroxide, superoxide, and hydroxyl radical are increased in diabetic patients and are commonly associated with cell damage. Moreover, it is well established that oxidative stress is an important metabolic abnormality in both cardiovascular disease and diabetic micro and macro-vascular complications (Nojima et al., 2008 ▶). As alternatives to chemical agents, plants are regarded as potential sources of antioxidants and hypoglycemic agents to control and treat diabetic patients (Patience et al., 2014 ▶; Malviya et al., 2010 ▶). Moreover, searching for new anti-diabetic drugs from natural plants is still attractive because of their low side effects, easy availability, roughly low cost, and also high effectiveness. *Urtica dioica L.* (*U. dioica*), a perennial herbaceous plant belonging to the Urticaceae family (Di Virgilio et al., 2014 ▶), is one of the medical herbs that has been traditionally consumed for a long time as medicinal plants in Iran and many parts of the world. Several studies showed beneficial effects of *U. dioica* against different diseases such as rheumatoid arthritis and diabetes (Nazemi et al., 2012 ▶). *U. dioica* has been extensively studied and has been promising in the treatment of prostate enlargement (Mamta and Preeti, 2014 ▶; Nahata et al., 2012 ▶), urinary tract infections and inflammation, nephrolithiasis, allergies, poor circulation, spleen enlargement, diabetes and other endocrine disorders. Also, *U. dioica* helps to lessen the swelling of hemorrhoids and stop bleeding from minor skin injuries (Ebrahimzadeh et al., 2015 ▶). It was also reported that *U. dioica* prevents the damage of liver tissue structure in rats (Turkdogan et al., 2003 ▶). In addition, *U. dioica* has been shown to have a protective effect against hepatic ischemia-reperfusion (Kandis et al., 2010 ▶), hyperglycemia (Otles and Yalcin, 2012 ▶) and hyper-cholesterolemia (Nassiri- asl et al., 2009 ▶). Supplementation of *U. dioica* leaves beverage has been shown to have a significant protective effect against TCA-induced liver injury (Celik and Tuluce, 2007 ▶). Moreover, some studies have shown antioxidant activity of *U. dioica* (Nazemi et al., 2012 ▶). Although, *U. dioica* is used for some *in vivo* and *in vitro* experiments, there is little evidence for the effect of *U. dioica* on diabetic complication and there is not any report about the effect of *U. dioica* on nitric oxide serum levels in diabetic patients. Therefore, the aim of the present study was to investigate the effects of hydro-alcoholic extract of *U. dioica* on some diabetes-related risk factors of cardiovascular incidence as well as oxidative stress biomarkers in patients with type 2 diabetes.

## Materials and Methods

A randomized double-blinded clinical trial was done on 50 women with type 2 diabetes (T2DM) in Diabetes Society of Shahroud affiliated to Shahroud University of Medical Sciences, Shahroud, Iran (IRCT2015031021412N1). The research was approved by the Ethics committee and Human Studies review board of Shahroud University of Medical Sciences by the identification code 920/11. The sample size was determined according to the following formula with consideration of type I error level α of % 0.05 and a test power of %90 (Khan et al., 2003 ▶).

n=2Z 1ɑ 2+Z 1β 22S2∆2

The sample size was computed as 20 per group. Regarding a possible loss to follow-up, a safety margin of 25% was determined, and therefore, 25 patients were allocated in each group. Participants were recruited from February 2014 to May 2014. The inclusion criteria to study were as follows: Women over the age of 50 years old, HbA1c levels equal or less than 10%, using common diabetes drugs (metformin and glibenclamide), patients with triglyceride levels less than 400 mg/dl. The exclusion criteria were: patients that have cardiovascular, chronic kidney and liver diseases, allergies and who were regularly using non-steroidal anti-inflammatory drugs (NSAIDs), warfarin, alcohol and insulin injection. Participants were divided into two groups: First, 25 women with type 2 diabetes who did not receive hydro-alcoholic extract of *U. dioica* (control group) and second, 25 women with type 2 diabetes who received hydro-alcoholic extract of *U. dioica* (intervention group). After being informed about the aim of present investigation, each patient signed an informed consent form, and was advised to continue her diet and physical activity habits without any changes during the intervention. After adjusting the patients by age and duration of diabetes, they were randomly divided into control and intervention groups using computer’s random numbers (Mahluji et al., 2013 ▶). 

**Figure 1:**
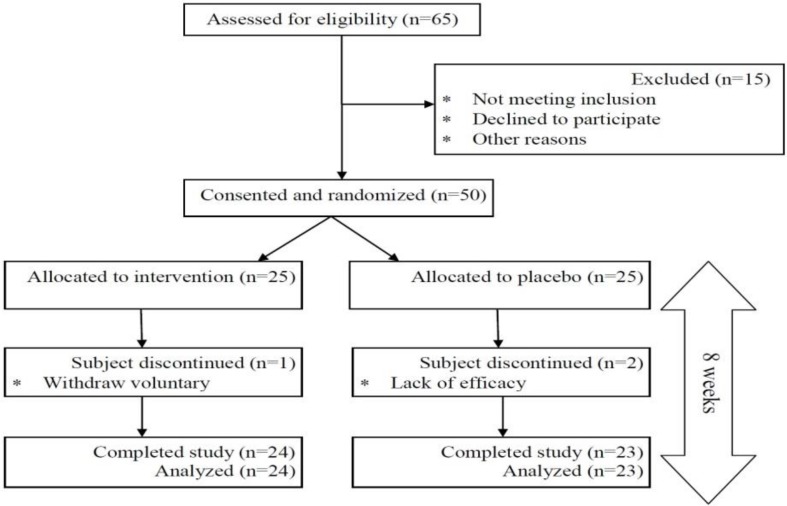
Study flowchart describing the progress of the trial

Patients received 5 ml of hydro-alcoholic extract of *U. dioica* or placebo (water, alcohol and chlorophyll color) in 3 portions a day (every 8 hr), after each main meal. Both experimental and placebo treatments were contained in the same bottles of liquid herbal extract with identical appearance, which were administered by a blinded research assistant. They dissolved each portion in 1 glass of water and continued for 8 weeks. The Participants in intervention and placebo groups were instructed to complete 24-hr dietary recall for three days (2 week days and 1 weekend day) at baseline and the end of study. Weight and height were measured by standard methods and the demographic characteristics (including BMI) were recorded for all patients. BMI was calculated by dividing weight (kg) by square of height (m). Patients were contacted every week and they were asked for any complaint about using *U. dioica* extract such as probable side effects including nausea and reflux and serious drug interactions with other drugs. Also, they were asked to return used bottles of *U. dioica* extract and placebo and receive new bottles. The flowchart in Figure 1 describes the progress of the trial.

**Extract specifications**

*U. dioica* certified by the Pharmacogenosy department of Gorgan University of Medical Science, Gorgan, Iran. Aerial parts of *U. dioica* were dried and powdered and the extract was prepared with percolation method using ethanol [60% (v/v) ethanol/water]. Final hydro-alcoholic extract of *U. dioica* contained 45% ethanol, 55% water and 2.7 g of dry matter in 1 l of extract. Water and alcohol percentage in placebo was equal to water and alcohol percentage in *U. dioica* extract, chlorophyll color was added to placebo. There was not any difference in the color between *U. dioica* extract and placebo.

**Biochemical measurements**

Before and after 8 weeks of continuous treatment, blood sample was taken from forearm vein of 2 groups (at the beginning and the end of the study) after overnight fasting (12-14 hr) for biochemical analysis of fasting plasma glucose (FPG), triglycerides (TG), low density lipoprotein (LDL), high density lipoprotein (HDL), glutamic pyruvic transaminase (SGPT), glutamic oxaloacetic transaminase (SGOT), superoxide dismutase (SOD) and nitric oxide (NO) using commercially available kits of Abcam (UK). HDL and LDL levels were analyzed using a Pars Azmoon kit (Pars Azmoon Co., Tehran, Iran).

**Statistical analysis**

Data are reported as Mean±SD. Statistical analysis was performed by Prism (Harvey, 2007 ▶). The normality of the distribution of variables was determined by the Kolmogorov-Smirnov test. Data with Abnormal distribution were converted to normal distribution by calculating logarithmic ratio. For variables with normal distribution, a paired t-test was used and comparison quantitative variables between groups were performed by Student’s t-test. A p<0.05 was considered significant for all variables.

## Results

**Figure 2:**
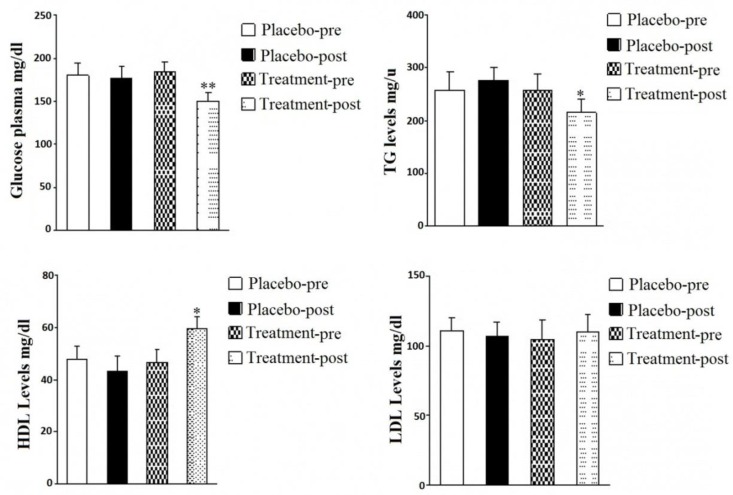
Effect of *U. dioica* hydro-alcoholic extract on plasma glucose level (A), TG level (B), HDL level (C), LDL level (D) in intervention group compared with placebo groups. *p < 0.05, **p < 0.01

In this study, 48 patients completed the study (24 patients of intervention and 24 patients of control group). Demographic and baseline characteristics of subjects including age, body mass index (BMI), duration of diabetes, physical activity, common diabetes drugs (metformin and glibenclamide), systolic and diastolic pressure, HbA1c and triglycerides level are described in Table 1. In the beginning of study, there were no remarkable differences in means of measured factors between the intervention and control groups. After treatment with hydro-alcoholic extract of *U. dioica*, the results showed that hydro-alcoholic extract of *U. dioica*, decreased fasting plasma glucose (Figure 2A) and TG level in intervention group at the end of 8-week treatment. However, an increase in TG level was observed in the patients using placebo (Figure 2B). The results showed that hydro-alcoholic extract of *U. dioica* increased HDL level in the intervention group after 8 weeks as compared to patients undergoing pretreatment with *U. dioica* extract (Figure 2C). The result showed that there were no significant differences in LDL level of intervention and placebo groups after 8 weeks (Figure 2D). Apart from glucose, the patients with type2 debates showed abnormalities in lipid metabolism as evidenced by significantly increased LDL levels which might contribute to various cardiovascular complications. 

**Table1:** Demographic and biochemical characteristics of type 2 diabetic patients in intervention and control group at baseline.

**Variable**	**Intervention** ** (n= 23)**	**Control** ** (n=24)**
**Age (Years)**	61.96±6.44	59.65±6.93
**BMI (kg/m** ^2^ **)**	23.56±2.22	23.28±1.96
**Duration of diabetes (Years)**	12.92±4.74	13.43±3.95
**Physical activity (%)**	65.30±9.6	63.12±10.5
**Metformin tablets (No/Day)**	2.33±1.48	2.52±1.51
**Glibenclamide tablets (No/Day)**	1.80±1.41	1.85±1.48
**Systolic pressure (mmHg)**	147.30±8.1	141.42±8.7
**Diastolic Pressure (mmHg)**	88.70±5.20	89.20±4.70
**HbA1c (%) **	7.80±1.60	8.10±1.9
**Triglycerides (mg/dl)**	256.10±29.6	255.63±36

Values are shown as Mean±SD.

**Figure 3:**
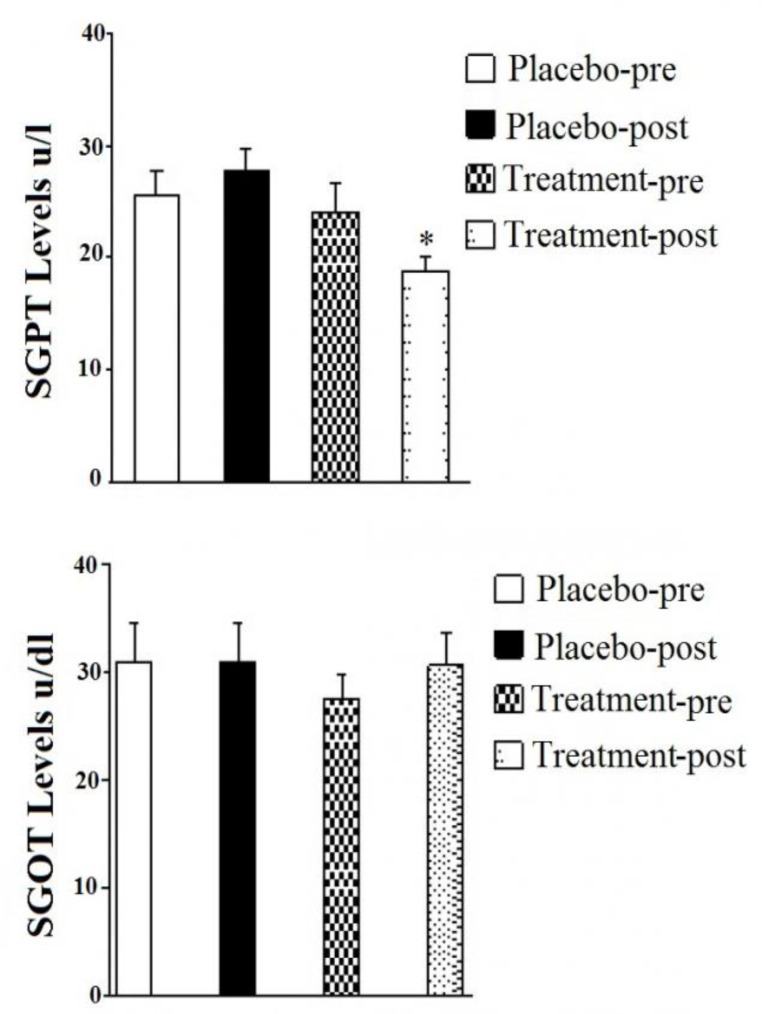
Effect of *U. dioica* hydro alcoholic extract on serum glutamic-oxaloacetic transaminase level, SGPT (A), serum glutamic pyruvic transaminase level, SGOT (B) in intervention group compared with placebo groups. *p < 0.05, **p < 0.01

In the present study, SGPT level was decreased in the intervention group with respect to the placebo group (Figure 3A). Patients undergoing post-treatment with *U. dioica* extract showed significant decrease (p<0.001) in SGPT after 8 weeks as compared to patients undergoing pre-treatment with *U. dioica* extract and patients using placebo. Further, there was no significant decrease in SGOT level in patients undergoing pre-treatment and post-treatment with *U. dioica* extract and placebo group after 8 weeks (Figure 3B).

**Figure 4:**
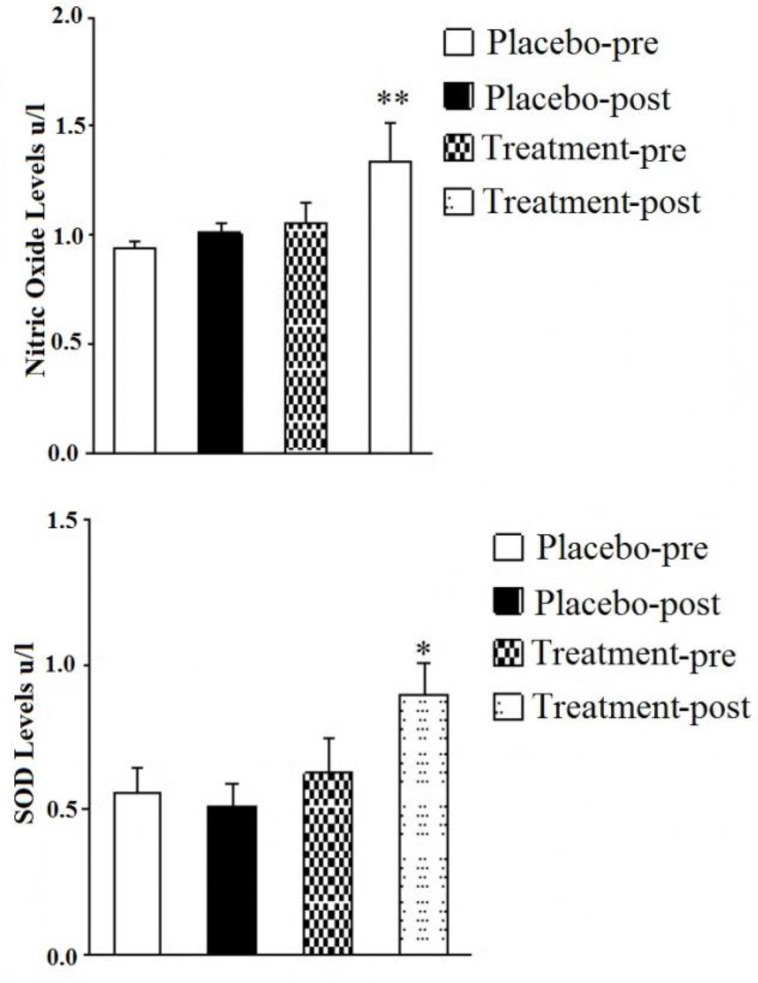
Effect of *U. dioica* hydro-alcoholic extract on serum levels of nitric oxide (A) and superoxide dismutase (B) in intervention group compared with placebo groups. ^*^p<0.05 and ^**^p < 0.01

Figure 4A shows that nitric oxide level (NO) was increased significantly (p<0.001) in patients undergoing treatment with *U. dioica* extract after 8 weeks as compared to placebo group. This study showed statistically significant differences (p<0.001) in SOD between the intervention and the placebo groups. The result showed that extract of *U. dioica* increased SOD in the patients undergoing 8 weeks of post-treatment as compared to patients undergoing pre-treatment with *U. dioica* extract at the beginning of the study and the placebo group (Figure 4B). 

## Discussion

Diabetes Mellitus is a clinical syndrome, characterized by hyperglycemia caused by a relative or absolute deficiency of insulin at the cellular level. It is the most common endocrine disorder, affecting mankind all over the world with an increasing prevalence (Karim et al., 2011 ▶; Tong and Cockrum, 2003 ▶). Traditional preparations from plant sources are widely used almost everywhere in the world to treat this disease (Patel and Udayabanu, 2013 ▶). *U. dioica* has shown a protective effect against hyperglycemia (Otles and Yalcin, 2012 ▶). Our results clearly indicate that the hydro-alcoholic extracts of *U. dioica*, decreases FPG level and potentially controls the hyperglycemic state of the patients with type2 diabetes after 8 weeks of treatment. The blood sugar lowering effect of *U. dioica* as a medicinal plant has been introduced before in animal models. Moreover, Farzami et al. (2003) ▶ showed that the increase in insulin level was associated with a decrease in FPG level. They reported that *U. dioica* extract can increase insulin secretion by Langerhans island and cause anti-diabetic and blood glucose-reducing effects. Current findings also showed that anti-diabetic property of *U. dioica* was due to its inhibitory effect on alpha-glucosidase. Furthermore, flavonoids can be effective in improving the blood glucose indexes via their antioxidant activity. Hydro-alcoholic extract of *U. dioica* could lead to rebuild beta cells in pancreas via its antioxidant characteristics (Golalipour and Khori., 2007 ▶). Moreover, tannin and carotenoids, as constituents of *U. dioica* could be effective in improving blood glucose indexes (Bahmani et al., 2014 ▶). Das et al. (2011) ▶ showed that *U. dioica* has an anti-hyperglycemic and anti-hyperlipidemic activity in type 2 diabetic rats. They also have shown that in the insulin deficient subjects, aqueous extract of *U. dioica* fails to activate the lipoprotein lipase enzyme, causes hypertriglyceridemia and lowers the cholesterol levels in type 2 diabetes model in rats. Therefore, a drug that is found to be active in type 2 diabetes models may have some role in decreasing cholesterol and triglycerides levels (Das et al., 2012 ▶). However, in our study similar to earlier studies on *U. dioica* (Pourahmadi et al., 2014 ▶), triglyceride levels deceased significantly whereas the decrease in concentrations of plasma cholesterol was not significant as compared with placebo group which may due to insufficient number of individuals. Pourahmadi et al. (2014) ▶ reported that *U. dioica* root extract decreased the HMG-COA reductase activity, resulting in lower plasma LDL levels in rats. Comparably, in our study, LDL level was increased in hydro-alcoholic *U. dioica* extract-treated group after 8 weeks. Moreover, high blood glucose causes fat deposits in the liver which is likely to increase SGOT and SGPT activities (Ahangarpour et al., 2014 ▶). Therefore, significant increase in SGOT/SGPT and decrease in HDL levels is observed in patients with type 2 diabetes. Similarly, in the present study, treatment of diabetic patients with *U. dioica*, reduced SGPT levels. Therefore, it is supposed that *U. dioica* could prevent liver damage in patients with type 2 diabetes. It is widely known that one of the most important vasorelaxing mechanisms depends on nitric oxide (NO), released from endothelium and acting through the stimulation of the soluble enzyme guanylate cyclase and elevation of the cGMP (Marazioti et al., 2011 ▶). In the present study, a decrease in NO level was observed in the patients using placebo and serum levels of NO were increased in patients after 8 weeks of treatment with *U. dioica* extract. Comparably, Tessari et al., (2010) ▶ and Ghosh et al., (2012) ▶ have shown that NO synthesis is reduced in subjects with type 2 diabetes and nephropathy. Moreover, Testai et al., (2002) ▶ concluded that *U. dioica* can produce hypotensive responses through a vasorelaxing effect mediated by the release of endothelial NO, opening of potassium channels, and a negative inotropic action in animal models. Golalipour and Khori, (2007) ▶ concluded that *U. dioica* has protective effect against oxidative stress in hyperglycemic rats. 

Toldy et al., (2005 ▶) showed that *U. dioica* (30 mg/kg) decreased ROS. Also in agreement with (Coskun et al., 2005 ▶) the present study showed that significant increase in the antioxidant enzyme activity of SOD in intervention group as compared with placebo group at the end of study. Phytochemical analyses of hydro-alcoholic extract of *U. dioica* showed that the polyphenols that are found in *U. dioica* include tannin, anthocyanin, chlorogenic acid, cafe oil malic, syringic, myricetin, quercetin, kaempferol, rutin, ellagic, isorhamnetin, p-coumaric, ferulic, naringin, fumaric, and vanillic (Otles and Yalcin, 2012 ▶). Phenolic compounds and especially a group of flavonoids which are present in *U. dioica* seem to be responsible for the antioxidant activity (Golalipour et al., 2011 ▶; Joshi et al., 2014 ▶; Khare et al., 2012 ▶) and play an important role in stabilizing lipid peroxidation (Bahmani et al., 2014 ▶). Quercetin 2-(3,4-dihydroxyphenyl)-3,5,7-trihydroxychromen-4-one, is dedicated to the greatest extent of flavonoids in *U. dioica*. These properties caused antioxidative characteristic of *U. dioica* in patients with type 2 diabetes (Jeong et al., 2012 ▶). Jeong et al., (2012) ▶ reported that consumption of quercetin can decrease plasma glucose levels and total cholesterol and increase HDL-cholesterol, hepatic GSH-Px activity and plasma adiponectin which is a hormone produced from adipose tissue and reduces insulin resistance. Our results showed that hydro-alcoholic extract of *U. dioica* is an interesting source of bioactive compounds and may decrease the diabetes-related risk factors of cardiovascular incidence and other complications in patients with diabetes mellitus. The main limitations of this study were the relatively small sample size, lack of precise control of diet and exercise of patients who participated in our study. Also, due to the relatively short duration of our study, we may have missed late beneficial effects and side effects of *U. dioica* extract; so, our results are suggestive rather than conclusive. Finally, similar studies with higher number of patients and longer duration of intervention are suggested for a better observation of the anti-oxidative effects of *U. dioica* in improving diabetic patient status.
